# Unusual Occurrence of Rare Lipid-Rich Carcinoma and Conventional Invasive Ductal Carcinoma in the One Breast: Case Report

**DOI:** 10.1155/2012/387045

**Published:** 2012-09-20

**Authors:** Katarina Machalekova, Karol Kajo, Marian Bencat

**Affiliations:** ^1^Department of Pathology, Cancer Institute of St. Elisabeth, Heydukova 10, 812 50 Bratislava, Slovakia; ^2^BB BIOCYT, Namestie L. Svobodu 1, 974 01 Banska Bystrica, Slovakia

## Abstract

A 56-year-old woman noticed a palpable mass in her left breast during self-examination. Patient was admitted to our hospital and malignant bifocal tumour was diagnosed by ultrasonography, digital mammography, magnetic resonance, and core-cut biopsy. The patient underwent planned conservative surgery (biquadrantectomy) with a sentinel node examination, but after results of the frozen section with positive resection margins and positive sentinel lymph nodes subsequent mastectomy with axillary lymph node dissection were realized. Histology in the resection specimen revealed two isolated and distinct tumours. One of the lesions represented conventional invasive ductal carcinoma of histological grade 3, and the second tumour was evaluated as invasive lipid-rich carcinoma, containing tumour cells with clear and foamy cytoplasm. Lipids in neoplastic cells were detected by Oil Red O staining and ultrastructural examination. Immunohistochemical analysis of both carcinomas was almost identical with negative steroid receptors, positive staining of HER-2, and p53 and with high proliferation activity (Ki-67). Mastectomy specimen contained residual foci of invasive ductal carcinoma and dissected axillary lymph nodes were free of metastasis. Patient underwent first cycles of chemotherapy with paclitaxel and Herceptin together with local radiotherapy and two month after surgery is without any evidence of the disease.

## 1. Introduction

Lipid-rich carcinoma of the breast is a rare subtype of the invasive ductal carcinoma (IDC) which has its own morphological code in the contemporary WHO classification [1] and is presented with an aggressive clinical course and poor prognosis [2]. This type of cancer comprises less than 1% of all breast malignant tumours [3]. Breast cancer is diagnosed as lipid-rich according to the neutral lipid substances that majority of carcinomatous cells contain in their cytoplasm. First case of this subtype of breast cancer was described by Aboumrad et al. in 1963 [4], who diagnosed carcinoma with lipid vacuoles in the neoplastic cells and designated the lesion as lipid-secreting carcinoma. Today is preferred term lipid-rich, describing only presence of the lipid substantives in the cells, unless there is an evidence of active lipid secretion in the neoplastic cells [5]. There are prevailing only sporadic case reports of lipid-rich carcinomas in the literature, and only several larger studies contained more patients (e.g., study of 49 cases by Shi et al.; analysis of 17 cases by Guan et al.) [2, 3]. 

Hereby, we represent a case of lipid-rich carcinoma in the coexistence with the second primary breast carcinoma in one mammary gland and we performed histological, immunohistochemical, and ultrastructural analysis of this rare type of breast cancer. 

## 2. Case Presentation

A 56-year-old woman visited our hospital in order to be examined for palpable mass in her left breast, which she noticed month ago. Patient during first visit underwent ultrasonographic examination and digital mammography, which disclosed two independent tumours, suspicious from malignancy, localized in the upper medial and upper lateral quadrant of the left breast. Skin of the breast above the lesions was clear and without any defects, nipple was without discharge. Subsequent core-cut biopsy from one of the lesions verified IDC. Results of the staging investigation, realized by computed tomography of the chest, abdomen, and bones, were normal. 

In the patient conservative surgery (biquadrantectomy) with sentinel lymph node biopsy was performed, followed by simple mastectomy with axillary lymph node dissection. Advanced surgery was realized after results of frozen section, which confirmed positive resection margins in specimen from quadrantectomy with finding of metastasis in two from three sentinel lymph nodes.

**Figure 1:**
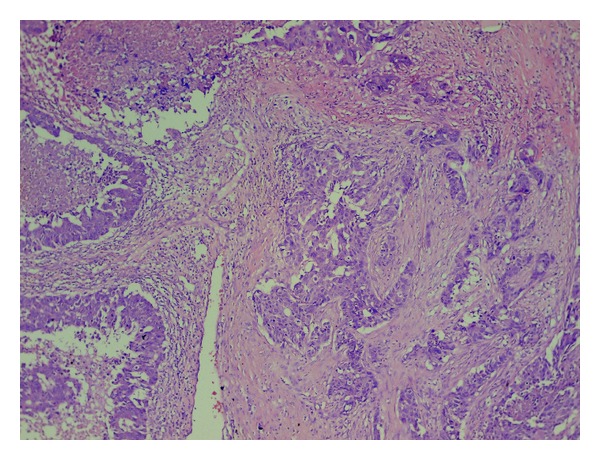
Larger tumour: conventional invasive ductal carcinoma of histological grade 3, with extensive necrosis and marked desmoplastic stromal reaction (hematoxylin-eosin stain, ×40).

**Figure 2:**
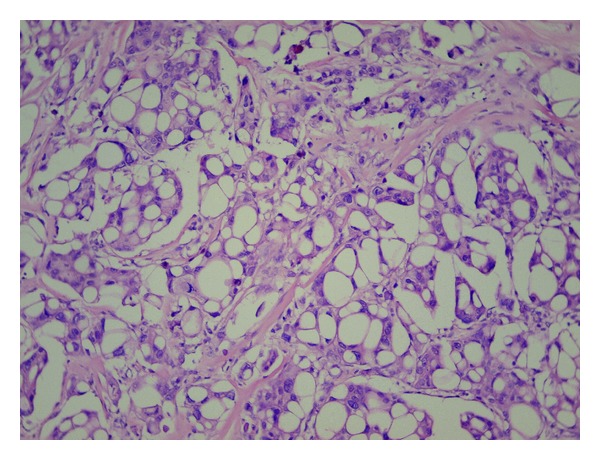
Smaller tumour: lipid-rich carcinoma of the breast, with atypical large vacuolated cells arranged in clusters, calcifications are seen in upper part of the picture (hematoxylin-eosin stain, ×200).

**Figure 3:**
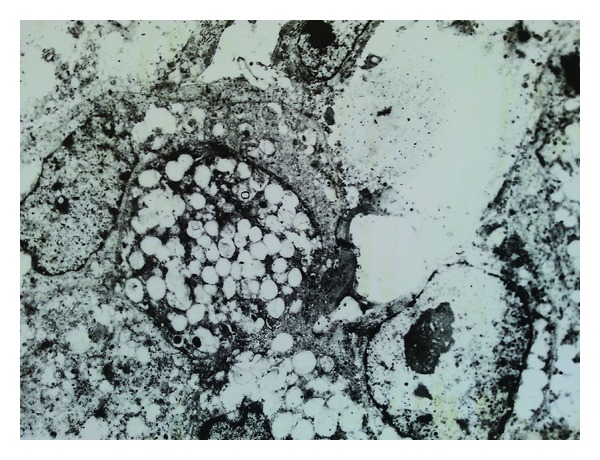
Electron microscopy: lipid-rich carcinoma cells with larger lipid vacuoles as well as with numerous lipid droplets in the cytoplasm.

**Figure 4:**
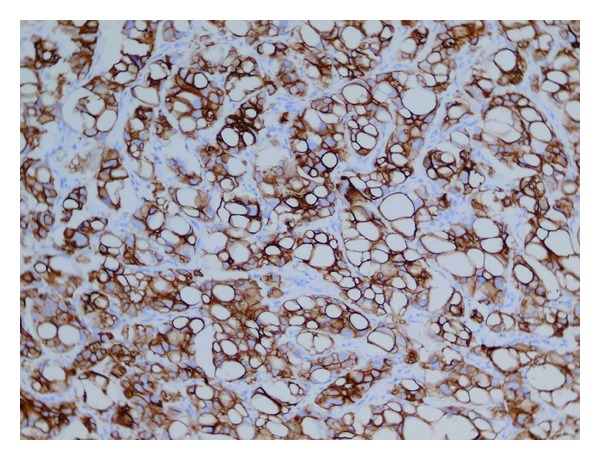
Lipid-rich carcinoma: immunohistochemical positivity of HER-2 (HercepTest Dako).

In the resection specimen there were found macroscopically two independent tumours, measuring 18 mm and 25 mm, and lying approximately 3 cm apart. Cut surface of the both lesions was distinct, smaller tumour was solid of yellowish colour, larger lesion contained cystic spaces with slurry staff inside. Microscopically, the larger tumour was diagnosed as conventional poorly differentiated IDC, with high degree of cytologic atypia, numerous mitotic figures, and widespread geographic necrosis as well as with signs of angioinvasion to the lymphatic vessels (Figure 1). The smaller neoplastic lesion was composed of large polygonal cells arranged in infiltrating solid and micropapillary formations, with abundant eosinophilic, vacuolated, and foamy cytoplasm (Figure 2). Neoplastic cells exhibited marked cytologic and nuclear atypia, many of the enlarged nuclei had conspicuous nucleoli. A lot of microcalcifications were present throughout the lesion. No in situ carcinoma component was observed. Foci of lymphovascular invasion were present in the adjacent breast tissue. In special staining on neutral lipids (Oil Red O), performed on formalin fixed, wet and unprocessed tissue, we detected positive reaction in nearly all vacuolated neoplastic cells. Periodic acid-Schiff (PAS) stain was negative. Also electron microscopy was realized, confirming many larger lipid vacuoles and smaller lipid droplets inside neoplastic cells. Vacuoles completely filled the cytoplasm of the cells and had marked membranes (Figure 3). Based on these findings we established diagnosis of invasive lipid-rich carcinoma of the breast, grade 3. Immunohistochemically, both carcinomas were negative for oestrogen (ER) and progesterone receptor (PR), cytokeratin 20, alpha-fetoprotein, HepPar-1 and WT-1, and were diffusely positive for HER-2 (Figure 4), p53, E-cadherin, cytokeratin 7, mammaglobin, and carcinoembryonal antigen. The proliferation activity, measured by Ki-67, was exceeding 20% in lipid-rich carcinoma and was over 80% in IDC. 

Two from three sentinel lymph nodes were positive with finding of metastasis of conventional IDC. The mastectomy specimen contained residual foci of typical IDC and all 18 dissected axillary lymph nodes were free of metastasis. 

The patient after surgery was without any complications, and in several weeks she started first cycle of the chemotherapy with paclitaxel and Herceptin, followed by local radiotherapy. Nowadays patient is two month after operation and is in a good shape, without any signs of the disease. 

## 3. Discussion

Lipid-rich carcinoma is a rare variant of the IDC that is histologically characterized by cells with numerous optically free vacuoles of various sizes in the cytoplasm. Age of the patients with lipid-rich carcinoma ranges from 33 to 81 years, and all except one were females [1]. Except of few reports that did not mention whether the carcinoma was invasive of intraductal, all reported cases of lipid-rich carcinoma were of invasive type, as it was present also in our case [5]. The definition of lipid-rich carcinoma is still not exactly established, because it is not clear, how many cells are supposed to be lipid containing to confirm the diagnosis [6]. Some authors suggested that lipid-rich carcinoma should contain lipid droplets in more than 90% of the neoplastic cells [1, 7, 8]. Also the origin of the lipids in the neoplastic cells is matter of discussion. Normal epithelial cells of the breast are able to synthesize not only proteins and carbohydrates, but also lipids [5]. Some authors believe that lipids in cells of the lipid-rich carcinoma are a secretory product of the malignant cells and not a result of degeneration. They support their theory by fact, that lipid vacuoles are in the cytoplasm localized close to the enlarged Golgi apparatus and huge endoplasmic reticulum, without finding of autophagic vacuoles [9, 10]. 

Lipid-rich carcinomas have distinct histological picture. Usually histology shows poorly differentiated invasive carcinoma, sometimes associated with a ductal or lobular in situ carcinoma. In situ areas of the lesion contain cells arranged in an alveolar pattern with a hobnail appearance. Invasive foci possess neoplastic cells, which are large and polygonal, with abundant vacuolated or foamy cytoplasm, full of neutral lipid and free of mucins or glycogen. Sometimes cells may confer a clear-cell or lipoblast-like appearance. The nuclei are fairly irregular, with moderate to severe atypia, usually containing one or more prominent nucleoli. Occasionally, large pleomorphic cells are arranged in alveolar pattern with a hobnail appearance, or they may show oxyphilic cytoplasm, reminiscent of oncocytic or apocrine change [11]. 

Due to the rarity of lipid-rich carcinoma, the association between immunohistochemical intrinsic subtypes and aggressiveness of the tumour has not been extensively studied. In a study of 17 lipid-rich carcinomas, all but one were negative for steroid receptors and all were HER-2 positive [3]. In other analysis of 49 cases of lipid-rich carcinoma, 5 of 49 (10, 2%) cases were ER+ and/or PR+ and 35 of 49 (71, 4%) cases exhibited HER-2 positivity [2]. Based on these observations, lipid-rich carcinomas are usually HER-2 positive with negative status of the hormonal receptors, as it was present also in our case. This tendency to high HER-2 expression in lipid-rich carcinomas can be responsible for worse prognosis and shortened disease-free survival period of the patients with this subtype of IDC [3, 12]. Some papers have been reporting the positivity of S-100 protein in lipid-rich carcinoma as a marker useful in its diagnostics [13]. Lipid-rich carcinoma usually shows a diffuse and intense immunoreactivity for lactoferrin and alpha-lactalbumin [14]. 

Ultrastructural analysis of lipid-rich carcinoma has been performed in small number of cases with divergent results. All authors described the presence of intracytoplasmic lipid droplets and globules isolated by distinct membranes and surrounded by a dense cytoplasmic rim [10]. Very similar observations were present in the electron microscopy realized in our case of lipid-rich carcinoma.

Differential diagnosis of lipid-rich carcinoma includes vacuolated or clear cell tumours of the breast, both of primary and secondary origin, mainly glycogen-rich carcinoma, apocrine carcinoma, secretory carcinoma, all of which contain some different metabolic products in their mainly foamy cytoplasm. Among the other tumours belong clear cell myoepithelioma and myoepithelial carcinoma, clear cell sarcoma, and metastatic renal cell carcinoma of clear cell type [5].

Glycogen-rich carcinoma contains PAS positive and diastase sensitive glycogen in the neoplastic cells. Moreover, glycogen-rich carcinoma possesses cells with more water-clear cytoplasm, whereas cells of lipid-rich carcinoma are more vacuolated and foamy [1, 10].

Apocrine carcinoma is a rare histological subtype of the breast cancer, composed of large pleomorphic cells with finely granular, eosinophilic cytoplasm and with large vesicular nuclei with prominent huge nucleoli [1, 10]. The cytoplasmic granules are commonly PAS positive and diastase resistant and neoplastic cells are consistently strongly positive for gross cystic disease fluid protein-15 (GCDFP-15). Moritani et al. presented their analysis of 26 apocrine carcinomas and 116 nonapocrine carcinomas, which all immunohistochemically stained for adipophilin, specific marker for lipids also in paraffin sections. They found intracytoplasmic lipids in 92% of all apocrine carcinomas and in 33% of the common breast carcinomas, where positive cell rate per tumor ranged from 10% to 70% in apocrine carcinomas. None of the apocrine and nonapocrine carcinomas had positive staining of adipophilin in more than 90% of the cells, thus the criteria for lipid-rich carcinoma was not fulfilled. Their immunohistochemical study suggested that lipid-rich carcinomas and apocrine carcinomas are closely related [7]. 

Secretory carcinoma, also referred as juvenile carcinoma, frequently occurs in younger women and unlike lipid-rich carcinoma has better prognosis. This subtype of carcinoma shows abundant intracellular and extracellular eosinophilic secretion and neoplastic cells contain PAS- and Alcian blue-positive substantives (mucopolysacharides) [14]. Secretory carcinoma has been recently shown to harbour the chromosomal translocation *t*(12; 15) (p13; q25), involving the genes *ETV6* and *NTRK3*, which appear to be specific for this subtype of breast cancer [15]. 

The positive immunostaining for myoepithelial markers (e.g., smooth muscle actin, CD10, high molecular weight cytokeratins, calponin, and p63) distinguishes myoepithelial tumours from lipid-rich carcinoma. Lipid-rich carcinoma may exhibit positivity for S-100 protein, but it is negative for the other myoepithelial markers [11]. 

The primary breast cancer is usually positive for mammoglobin and GCDFP-15, which are negative in metastatic clear cell carcinomas or sarcomas (e.g., renal cell carcinomas, where is positive staining for vimentin and CD10). 

Regardless of the small number of cases of lipid-rich carcinoma reported, the prognosis of this type of cancer seems to be very poor [4, 11], with 2- and 5-year overall survival rates 64, 6% and 33, 2%, respectively [2]. Many of the patients with lipid-rich carcinoma reported had nodal metastasis in the time of presentation and developed distant metastasis in next two years [11]. 

In conclusion, until now we do not have knowledge about publishing of lipid-rich carcinoma occurring with the second primary breast carcinoma in one mammary gland in the world literature. We regard this phenomenon as extremely rare, considering lipid-rich carcinoma of the breast as uncommon neoplasm itself. According to the known poor prognosis of lipid-rich carcinoma, it is necessary to make a correct diagnosis of this very rare neoplasm, which has different biopathological profile from the other types of the breast cancer. Early diagnosis of lipid-rich carcinoma and active oncologic treatment may be helpful to increase its overall survival.
